# A Bibliometric Review of Artificial Extracellular Matrices Based on Tissue Engineering Technology Literature: 1990 through 2019

**DOI:** 10.3390/ma13132891

**Published:** 2020-06-27

**Authors:** Pilar Simmons, Taylor McElroy, Antiño R. Allen

**Affiliations:** 1 Division of Radiation Health, University of Arkansas for Medical Sciences, Little Rock, AR 72205, USA; PGSimmons@uams.edu (P.S.); TMMCELROY@uams.edu (T.M.); 2 Department of Pharmaceutical Sciences, University of Arkansas for Medical Sciences, Little Rock, AR 72205, USA; 3 Department of Neurobiology & Developmental Sciences, University of Arkansas for Medical Sciences, Little Rock, AR 72205, USA

**Keywords:** artificial extracellular matrix, tissue engineering, biomaterials, regeneration

## Abstract

Artificial extracellular matrices (aECMs) are an extension of biomaterials that were developed as in-vitro model environments for tissue cells that mimic the native in vivo target tissues’ structure. This bibliometric analysis evaluated the research productivity regarding aECM based on tissue engineering technology. The Web of Science citation index was examined for articles published from 1990 through 2019 using three distinct aECM-related topic sets. Data were also visualized using network analyses (VOSviewer). Terms related to in-vitro, scaffolds, collagen, hydrogels, and differentiation were reoccurring in the aECM-related literature over time. Publications with terms related to a clinical direction (wound healing, stem cells, artificial skin, in-vivo, and bone regeneration) have steadily increased, as have the number of countries and institutions involved in the artificial extracellular matrix. As progress with 3D scaffolds continues to advance, it will become the most promising technology to provide a therapeutic option to repair or replace damaged tissue.

## 1. Introduction

The field of tissue engineering has an extensive range of potential applications in tissue repair and regeneration [1]. The role of biomaterials in tissue engineering is to provide support and scaffolding for cell growth, one of the principal factors that determine the success of tissue regeneration [1,2,3]. The utilization of biomaterials as a possible replacement, maintenance, and/or repair for damaged or diseased tissues in a living system is a prime objective in the study of biomaterial research [4,5,6]. The number of publications on tissue engineering using the biomaterial approach continued to increase in recent decades. Tissue engineering is a rapidly advancing field that relies on scaffold biomaterials to provide the appropriate environment to guide the growth of new tissue [7,8,9]. The extracellular matrix (ECM) encompasses the natural surroundings of cells in vivo that not only maintain structure but regulate many aspects of cell behavior, including cell proliferation, growth, and differentiation [10,11]. Cellular differentiation is a broad process that aims to specialize a cell in order to perform specific function [12,13,14]. Cell-matrix interactions are pivotal in differentiation regulation and development [15,16,17]. The construction of a template ECM surface with the capability of providing a pertinent, in-vitro environment to regulate cellular behavior is a leading approach in biomaterial publications [7,10,18].

Artificial extracellular matrices (aECMs) are an extension of biomaterials that were developed to provide a model environment for tissue cells in-vitro that mimic the native surroundings of the target tissue in vivo [10,18,19]. The structure of native ECM is contingent upon the developmental stage and type of tissue; therefore the architecture and components of an aECM vary [10,18]. The optimal fabrication techniques used to construct aECMs are a focus of many tissue engineering studies and frequently presented in publications. The composition of each aECM is constructed to support the cellular processes for the optimal function of the target tissue [6,18,20]. Artificial ECMs are generally classified as either a natural polymer-based, synthetic polymer-based, or hybrid material, and they differ based on their component materials [18,20]. Over the years, the use of aECMs has attained an excellent reputation in the field of tissue engineering due to their exceptional mechanical properties, processability and low cost [21,22]. The ideal aECM is physiochemically similar to the target tissues innate ECM, non-toxic, biodegradable, affordable, durable and poorly immunogenic [23,24]. Both naturally derived and synthetic materials have been developed to mimic native ECM for the study of regenerative medicine and tissue engineering [21,25]. Materials from natural sources such as laminin, fibronectin and vitronectin are beneficial to cell culture because they contain cell recognizable receptors that enhance cell-to-matrix interactions [26]. However, there are some challenges with natural materials such as the risk of pathogen transmission, purification, immunogenicity and structural complexity [21,27]. Advancements in synthetic biomaterials are being developed at a rapid pace for use as 3D extracellular microenvironments to mimic regulatory components and functions of natural ECMs [21,28]. Novel designs of aECMs from synthetic materials are a possible alternative due to their minimal risk of pathogen transmission, lack of immune response and the ability for greater control over material properties and tissue responses [21,29]. The type of target tissue, accessibility and the biological or therapeutic application of the study are all a few factors to take into consideration when choosing between the various biomaterials available.

The significant attraction in the study of artificial ECMs is largely attributed to their potential applications in the field of regenerative medicine [26,30,31]. There is a considerable amount of studies that focus on the use of aECMs and biomaterials to treat tissue damage and promote tissue regeneration. There has been a recent interest in biomaterial-based approaches to treat the lack of regeneration from spinal cord injuries (SCI) [32,33]. A leading factor in the permanence of SCI is the inability of the damaged axons to regenerate, preventing proper function of axonal circuits [34,35]. The emergence of biomaterials for regeneration has increased collaborations between engineers, scientist and clinicians to design materials that address the specific criteria for repairing this type of injury [34,36]. Tissue damage to skin, bone, liver, lung, heart and the vascular system are among a few targets that also utilize aECM strategies to study potential treatment and repair [32,37,38]. With increasing cases of organ shortages and donor scarcity within the last three decades, the research focus in the field of regenerative medicine and tissue engineering continues to advance toward a potential therapeutic for various types of tissue damage [39,40].

Many reviews highlight the current strategies and future prospects of aECMs in tissue engineering; however, there is a lack of bibliometric reviews on the research output of publications. We aimed to evaluate and document how this field is expanding. We assessed the literature on aECMs based on tissue engineering over three decades by performing a bibliometric analysis that highlights (1) the most common terms occurring in related papers and (2) research volume output by authors, institutions, countries, and journals and their impact.

## 2. Materials and Methods

### 2.1. Data Source and Search Strategy

Data were obtained from the Web of Science online database (Clarivate Analytics, Philadelphia, PA, USA) in January 2020. Web of Science is a universal scientific citation index used to retrieve scholarly articles and academic literature [41,42]. This bibliometric study analyzed articles related to major journals publishing aECM research from 1 January 1990 through 31 December 2019. Three separate topic sets of search terms were employed in an “advanced search”: (Topic set = (insert topic terms)), language: (English), and document types: (Article or Abstract or Published Item or Review)). The key search terms were categorized into three separate topic sets:

(1) Topic set 1 = (tissue engineer and regeneration);
(2) Topic set 2 = (biomaterial and regeneration);
(3) Topic set 3 = (artificial extracellular matrix) and (stem cell or axon or neural regeneration or central nervous system or regeneration or tissue engineer or biomaterial).

The Web of Science Core Collection Citation Indexes: Science Citation Index Expanded, Social Sciences Citation Index from 1990 through 2019 was applied to all three topic sets. The “custom year range” timespan of 1990 through 2019 was subdivided into three time periods (1990s, 2000s, and 2010s) for each set to analyze temporal trends. Book chapters, proceedings papers, and retracted publications were excluded from the results of all three topic sets. Web of Science citation data extracted for each topic set and selected time period were downloaded as full records and cited references and saved in a tab-delimited file format. The data that were downloaded included publication by institution, publication by country, publication by funding agency, and publication by author.

### 2.2. Data Analyses and Presentation

The extracted citations were imported into VOSviewer version 1.6.14 (Centre for Science and Technology Studies, Leiden University, Leiden, The Netherlands) [43] for the network analyses. Throughout the VOSviewer data importation and analysis, the topics were separated into three separate sets and three time periods (1990s, 2000s, and 2010s) as mentioned in the Web of Science searches. The term maps generated for software analysis were selected using the following alternatives: “create a map based on bibliographic data”, “read data from bibliographic database files”, “type of analysis: Co-occurrence”, “unit of analysis: all keywords” and “counting method: full counting”. When a bibliometric map is created, it is often helpful to create a thesaurus file that can merge terms, correct spelling differences, or exclude general terms. A list of the top 5000 words from the 450 million word Corpus of Contemporary American English was downloaded and used as a thesaurus to exclude these terms from the analysis [44]. The list was altered with terms such as extracellular-matrix and extracellular matrix so that the software would recognize them as the same term. Applying these settings allows the software to identify words in titles and abstracts of publications as they relate to papers in which they occur together. After the terms are analyzed by the software, they are arranged in a network of keywords that are projected as a collection of bubble figures. Each bubble represents a word or phrase that is sometimes referred to as an item. The color of the bubble relates to the cluster it belongs to and the size of the bubble represents the frequency of the term based on the occurrences in the publications. The larger the bubble appears, the higher the number of occurrences it represents. If a term appears in a single publication multiple times, it is still counted as one occurrence in the data analysis. The distance between each bubble relates to the co-occurrence of terms in publications. The closer the bubbles are to one another, the higher the number of co-occurrences. The minimum occurrence for keywords varied depending on the three time periods: 1990 through 1999 (1990s), 2000 through 2009 (2000s), and 2010 through 2019 (2010s). As the publications increase over time, the occurrences were increased as well. In the 1990s, the minimum occurrences of a keyword ranged from three to five; in the 2000s, the minimum occurrences of a keyword ranged from 8 to 15, and in the 2010s, the minimum occurrences of a keyword ranged from 13 to 35. The calculation of “averaged citations” was also selected to display the average number of citations received by the documents in which a keyword or a term occurs. Lastly, maps illustrating the countries’ data for each of the topic related papers from 1990 through 2019 was created with Excel 2016 (Microsoft, Redmond, WA, USA).

## 3. Results

### 3.1. Publication Output: Topic Set 1, 2 and 3 for 1990–2019

**Figure 1:**
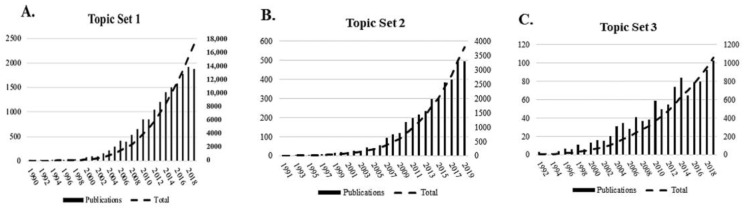
Number of publications and cumulative number of publications for topic set 1(**A**), topic set 2(**B**) and topic set 3(**C**) per year.

The output of research related to topic set 1 of our terms search, in the form of publications, increased continuously from 1990 through 2019, resulting in a total of 17,082 publications. The first paper was published in 1990 and publications reached double-digit numbers per year in 1994. Since 2012, over 1000 papers have been published per year. (Figure 1A). The output of research related to topic set 2 of our terms search, in the form of publications, also increased continuously from 1991 through 2019, resulting in a total of 3801 publications. The first paper was published in 1992, and yearly publications reached double digits with 16 publications in 1999. In 2007, the number of publications finally exceeded 100. Publications continued to increase into the 2010s but did not reach above a triple-digit number per year (Figure 1B). The publication output of research on aECMs related to topic set 3 of our terms search increased overall from 1991 through 2019, with a minor drop in publications in 1999, resulting in a total of 1057 publications. The first paper was not published until 1992 with only three publications. Papers on aECMs did not reach double digits until 1998, and it did not reach the triple digits until 201 with 103 publications. (Figure 1C). The gradual increase in publications in each of the topic sets is further indicated by the cumulative number of publications (Figure 1A−C).

### 3.2. Evolution of Bibliographic Terms: Topic set 1 for the 1990s and 2010s

**Figure 2:**
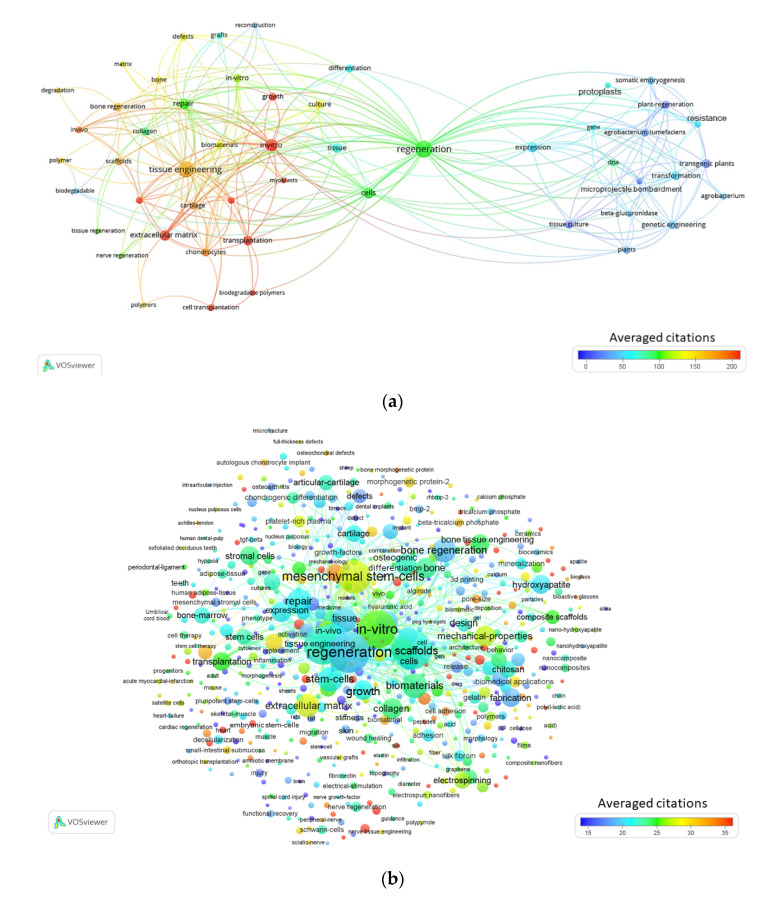
Term map for years (**a**) 1990 through 1999 (**b**) 2010 through 2019. (**a**) Term map showing the visualization of 51 terms. Table S1 contains all the terms visualized with their respective occurrence frequencies and averaged citations. (**b**) Term map showing the visualization of 630 terms. Table S3 contains all the terms visualized with their respective occurrence frequencies and averaged citations.

The term maps generated from VOSviewer from topic set 1 are displayed in Figure 2a,b according to their perspective time periods (1990s and 2010s). The results from the 1990s consisted of 51 terms with three clusters, 494 links, and a total link strength of 947 (Figure 2a). The terms with the highest number of occurrences besides regeneration and tissue engineering were extracellular matrix and repair. By observing the maps, it is apparent that the publications connected to the terms tissue engineering and regeneration were very general in the first decade of exploring the field. Common links to the terms appearing in tissue engineering and regeneration-related papers were basic terms such as polymer, scaffolds, collagen and biomaterials. There was also a distinct cluster of terms that indicate an interest in the study of connections between regeneration and botany with an occurrence of terms such as protoplasts, somatic embryogenesis, agrobacterium tumefaciens and transgenic plants. The results from the 2010s consisted of 630 terms with six clusters, 74,852 links, and a total link strength of 432,343 (Figure 2b). The terms with the highest occurrences besides regeneration and tissue engineering were scaffolds, in-vitro, biomaterials, extracellular matrix, and mesenchymal stem cells. In comparison with the 1990s, the detail of biomaterial related occurrences increased significantly in the 2010s, with terms such as alginate, chitosan, hydrogels, nanocomposite and hylauronic acid. Studies on the role of tissue engineering and regeneration in the potential application of tissue repair started to appear, with key term occurrences such as spinal cord injury, wound healing, axonal regeneration, bone regeneration, cardiac regeneration and cartilage regeneration.

### 3.3. Evolution of Bibliographic Terms: Topic Set 3 for the 2010s

**Figure 3:**
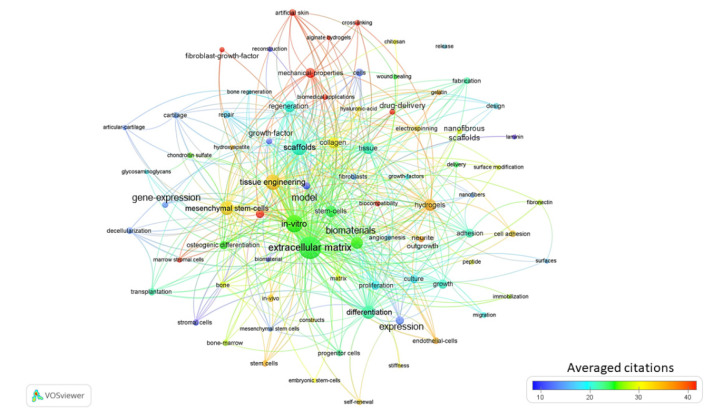
Term map for years 2010 through 2019. Term map showing the visualization of 79 terms. Table S9 contains all the terms visualized with their respective occurrence frequencies and averaged citations.

The term map generated from VOSviewer using topic set 3 are displayed in Figure 3 according to the 2010 through 2019 time period. The results from the 2010s consisted of 79 terms with five clusters, 1887 links, and a total link strength of 6996 (Figure 3). The terms with the highest occurrences were hydrogels, mesenchymal stem cells, and regenerative medicine. Hydrogels are a network of polymer chains that were commonly mentioned in a broad number of publications that discussed the design of aECMs. It is not unexpected that mesenchymal stem cells had a high occurrence, seeing as a lot of publications related to aECM focused on the possible treatment of bone injury. The term in-vivo had an occurrence of 32, with 46 links and a total link strength of 105. During the 2010s, there was an increase in addressing the feasibility of applying aECM studies to clinical applications.

### 3.4. Evolution of Bibliographic Terms in Relation to Countries of Origin: Topic Set 1

**Figure 4:**
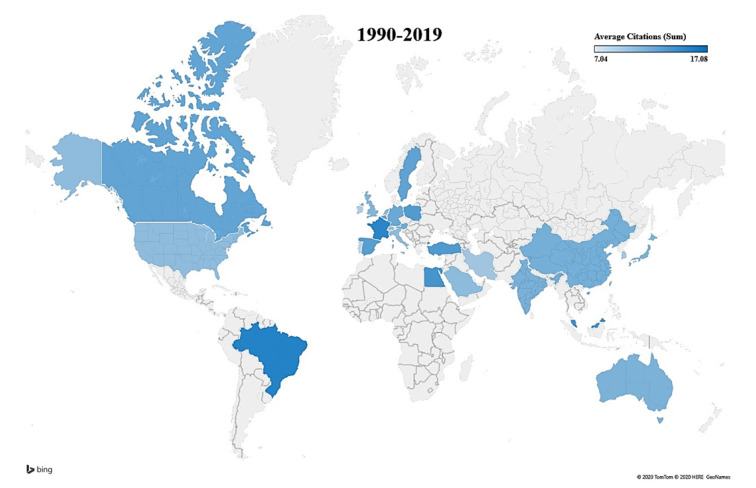
World map representing the averaged citations per topic set 1 related papers published 1990 through 2019. Papers may have authors from more than one country denoting international collaboration. Please refer to the color scale for averaged citations. Countries with at least 20 publications are represented.

Between 1990 through 1999 papers about topic set 1 originated mainly in the USA followed by England (Table 1). During this time period, the USA was also the leading producer of citing articles. Between 2000 through 2009, USA continued as the top-ranking country and China followed behind as the 2nd rank. This trend continued from 2010 through 2019. Research into topic set 1 is worldwide and a large volume of publications originate from USA with China following closely behind (Figure 4). 

### 3.5. Evolution of Bibliographic Terms in Relation to Countries of Origin: Topic Set 2

Similar to topic set 1, papers about topic set 2 originated mainly in the USA, followed by England in the 1990s (Table 2). The USA was also the leading producer of citing articles (1145). This continued throughout the 2000s and the 2010s. By the 2000s, China ranked 2nd in both total publications and citing articles. The USA and China continued to dominate the publication output over the course of three decades.

### 3.6. Evolution of Bibliographic Terms in Relation to Countries of Origin: Topic Set 3

Between 1990 through 1999, papers about topic set 3 originated mainly in the USA, followed by Germany (Table 3). Japan, France and Italy also published a sizable amount of research. During this time period USA was the leading producer of citing articles (690). The USA produced twice the amount of Germany (312) while China, Italy and Japan followed relatively close behind. Between 2000 through 2009, USA was still the top-ranking country for publications and Japan replaced Germany as the 2nd rank. The USA also continued to rank first in production of 7132 citing articles. From 2010 through 2019, the USA continued to lead as the number one country, with China emerging as the 2nd rank.

### 3.7. Profile of the Top 10 Most Productive and the Most Cited Authors: Topic Set 1 for 1990 through 2019

Throughout this time period, Reis R.L. ranked first with 195 publications, followed by Ramakrishna S. with 141 publications (Table 4). Wang Y. was the top cited author with 1699 total citing articles. Wang L., Wang J. and Lee S.J. were all in the lead with an average of 15 citations per publication.

### 3.8. Profile of the Top 10 Productive and the Most Cited Organizations: Topic Set 1 for 1990 through 2019

Throughout this time period, the most productive organization in terms of publication output was Harvard University with 446 total publications (Table 5). The University of California System are the leaders for the top citing articles at 6018. However, the Chinese Academy of Sciences had the most citations per publication with about 17 citations. Shanghai Jiao Tong University follows closely behind as the top producer, with a total publication count of 440. American and Chinese institutions are the leading producers for publications on topic set 1.

## 4. Discussion

Bibliometric analysis is a tool used to quantitatively evaluate the output of scholarly journal publications and measure the significance of studies in the scientific community [44,45,46]. This is a very different approach to scientific writing than a review of the literature that summarizes a specific topic from various publications [47]. Citation data are key to assessing the academic influence of a publication by the number of times it has been cited by other authors [44,45]. Results from the bibliometric analysis can also provide information that influences science policymakers and research funders’ decisions [44,46]. The analysis can also be a method of ranking journals, institutions, and universities worldwide [48]. This bibliometric analysis aimed to evaluate the research productivity of aECMs based on tissue engineering technology. The database search was performed through Web of Science citation index on articles published from 1990 through 2019, with titles pertaining to aECM, tissue engineer, biomaterial, and regeneration.

The field of TE-related research is continuously growing, as presented in the rising number of publications and the expansion of research areas involved. TEs’ in-vitro research is gradually being transitioned to being applied to in vivo studies with a more clinical focus. This trend is reflected in the last decade as more terms gradually appear to be related to clinical topics like biocompatibility, grafts, transplantation, wound healing, nerve regeneration and bone regeneration. This is evident in the term maps of the most recent publications that highlight groups of tissue regeneration terms that overlap in several publications (Figure 3, Table S9). The current dependence on donated tissue and organs is not the most feasible approach as the population continues to age and as the scarcity of organ donors increases [49]. TE and regenerative medicine (RM) is a promising method to address the urgent demand for organs and tissues needed for transplantation [50]. In the 2000s for topic set 1 there were quite a few terms that related to bone and cartilage such as chondrogenic differentiation, bone morphogenetic proteins, chondrogenesis, bone tissue engineering, bone formation, articular-cartilage, bone marrow stromal cells and osteogenic differentiation (Figure S1, Table S2). For topic set 2, from the 1990s to the 2000s there was a significant increase in publications and studies as indicated by the extensive expansion of key terms from 25 to 102 (Figure S2, Tables S4 and S5).

Biomaterials are important components of tissue engineering studies as shown in the term maps generated for topic set 1. The appearance of terms such as biodegradable polymer, hydrogels, scaffolds, collagen, alginate and fibrin were shown throughout the time period, with scaffolds appearing consistently (Figure 2a,b, Tables S1 and S3). During the early decades of the topic set 3 searches the biomaterial related key words were general and extremely vague (Figure S3, Tables S7 and S8). Advancements in biocompatible materials into complex 3D tissue models provide considerable opportunities for translational research that is slowly moving toward clinical applicability [49]. There are a few engineered tissues that are being transitioned toward a pre-clinical phase, with the potential to be implanted into patients, such as bladder, skin, trachea, vascular grafts, cartilage and bone [49,51,52]. There were several terms on the 2010 through 2019 maps that appear in publications that relate to these tissues such as mesenchymal stem cells, vascular grafts, stromal cells, bone marrow, chondrocytes and artificial skin (Figure S2, Figure 2b and Figure 3). 3D scaffolds and matrices arranged to mimic the native micro-environment and biological components within the body are continuing to evolve and advance over time. However, there are still limitations and challenges that need to be settled before they can be considered suitable for clinical application. There are a number of hurdles that raise concerns, such as ensuring that materials are harmless to the host and that they support the composition and function of the cells, immune complications, and spatial constraints [49,53]. The ECM and cellular composition of different tissues vary widely between cell types and they each pose their own engineering challenges [49,54]. It is vital for biomaterials to not only mimic the structural components of the ECM, but to also have the ability to promote adhesion, differentiation and proliferation of cells [49,55,56]. Over the last three decades, there has been an increase in publications developing various types of biomaterials as shown in the key term maps for topic set 2. Hydroxyapatite, chitosan, hydrogels, nanofibers, keratin and alginate are examples of common occurrences in the 2000s and 2010s (Figure S2 and Table S6). It is evident that understanding the fundamental biology of tissue formation and morphogenesis is important [24,30]. There are also some limitations to using human models when moving towards the clinical application that could cause concern for legal constraints. However, the design of ideal biomaterials that can successfully interact with cells within tissues after implantation is a challenge that continues to be studied in TE technology [21]. 

Regarding the global trend for topic set 3, there was a large output of publications from the USA. Japan and Germany also contributed a substantial amount of publications related to topic set 3 throughout the time period. The extensive number of publications from these countries were reflected in the large number of articles that cited these publications. For topic set 2, the USA and China were leading in both publication output and citing articles. However, countries like South Korea and Germany were not consistently the top producers of publications, but they did contribute a considerable number of citing articles. For topic set 1, the USA was again the lead producer of publication output throughout the entire period. In the 1990s, China was not a top publication producer, but that shifted in the 2000s and the 2010s as their publications begin to increase. Both countries also continued to lead in citing articles throughout each decade. Although the USA produced the most research for each topic set, their relative contribution to the percentage of the total global output decreased as the other counties began to maintain a significant contribution to their publication output each decade.

Many of the top authors results for each of the topic sets identified individuals that came from the same lab or collaborating labs. A considerable amount of the top authors also coincided with the top country results. For topic set 3, the primary rank for publication output continued to shift throughout each decade. Yannas and Minuth remained in the top five producing authors until the last decade, when all new authors took the lead. Authors of the top citing articles varied greatly from the authors with the greatest publication output. However, there could be a possibility that some of the top producing authors collaborated on publications with the authors from the top citing articles. This trend continued into publications related to topic set 2, aside from Kaplan, Reis and Mikos, who appeared in the top rank for both the publication output and the citing articles at least once in each decade. For topic set 1, quite a few of the top producing authors concur with the authors from the top citing articles throughout the time period. 

The institutional results for topic set 3 correspond with the results from the top producing countries based on the geographical location of the institution. For topic set 3 in the 1990s and the 2000s, the USA was the leading producer and citer of publications. This aligns with the results of American institutions such as MIT, Harvard University and the University of California System leading in publication output and citing articles. In the 2010s, Dresden University of Technology in Germany was the leading institution for publications and the Chinese Academy of Sciences in China was the leading citer of publications. This also coincides with the data from the countries during that time period. For topic set 2, during the entire time period the USA and the China continued to the lead the top charts for both publications and citing articles. For topic set 1, the American institutions dominated the publication output and the citing articles for the first two decades. In the 2010s, Chinese organizations such as Shanghai Jiao Tong University were the top producers and the top citer of publications was the Chinese Academy of Sciences (Table 4).

## 5. Conclusions

In conclusion, this analysis demonstrated that the artificial extracellular matrix literature based on tissue engineering technology is growing exceptionally fast in research related to biomaterials and regenerative medicine. It is expected that we will continue to see an increasing research output in the near future as 3D scaffolds continue to advance their design to resemble the natural ECM as closely as possible. As progress with 3D scaffolds continue to advance it will become the most promising technology to provide a therapeutic option to repair damaged tissue.

## Figures and Tables

**Table 1:** Most productive and influential countries throughout time for Set 1.

**1990–1999**			
**Rank**	**Country**	**TP**	**TC**
1	USA	96	6203
2	England	11	1166
2	Japan	11	1400
3	Germany	10	1289
4	Canada	7	680
**2000–2009**			
**Rank**	**Country**	**TP**	**TC**
1	USA	1149	30,299
2	China	306	21,492
3	Japan	298	7655
4	Germany	234	6950
5	England	201	6252
**2010–2019**			
**Rank**	**Country**	**TP**	**TC**
1	USA	3901	24,697
2	China	3549	24,624
3	South Korea	1068	5574
4	Italy	850	5143
5	Germany	827	6351

The ranking is based on the number of total publications; TP = Total publications; TC = Total citing articles.

**Table 2:** Most productive and influential countries throughout time for Set 2.

**1990–1999**			
**Rank**	**Country**	**TP**	**TC**
1	USA	21	1145
2	England	7	222
3	France	6	177
4	Netherlands	5	120
5	Italy	4	249
**2000–2009**			
**Rank**	**Country**	**TP**	**TC**
1	USA	191	8527
2	China	50	6576
3	Japan	49	1794
4	Italy	46	1827
5	Germany	43	2395
**2010–2019**			
**Rank**	**Country**	**TP**	**TC**
1	USA	857	9428
2	China	656	10,505
3	Germany	247	2668
4	Italy	240	2402
5	South Korea	193	2325

The ranking is based on the number of total publications; TP = Total publications; TC = Total citing articles.

**Table 3:** Most productive and influential countries throughout time for topic set 3.

**1990–1999**			
**Rank**	**Country**	**TP**	**TC**
1	USA	15	690
2	Germany	7	312
3	Japan	5	210
4	France	3	74
4	Italy	3	221
**2000–2009**			
**Rank**	**Country**	**TP**	**TC**
1	USA	91	7132
2	Japan	46	1494
3	Germany	36	1717
4	South Korea	17	1368
5	England	15	1349
**2010–2019**			
**Rank**	**Country**	**TP**	**TC**
1	USA	206	4123
2	China	117	3635
3	Germany	95	1201
4	Japan	87	704
5	South Korea	63	930

The ranking is based on the number of total publications. TP = Total publications; TC = Total citing articles.

**Table 4:** The top productive and most cited authors of topic set 1 for 1990 through 2019.

Rank	Combined 1990–2019			
	Authors	Publications	TC	CPP
1	Reis R.L.	195	1201	6.16
2	Ramakrishna S.	141	576	4.09
3	Wang Y.	134	1699	12.68
4	Liu Y.	127	1606	12.65
5	Zhang Y.	111	1632	14.70
6	Kaplan D.L.	98	861	8.79
7	Wang J.	89	1343	15.09
8	Wang L.	87	1343	15.44
9	Lee S.J.	83	1285	15.48
10	Boccaccini A.R.	81	871	10.75

The ranking is based on the number of total publications; TP = Total publications; TC = Total citing articles; CPP = Citations per publication.

**Table 5:** The top productive and most cited institutions of topic set 1 for 1990 through 2019.

Rank	Institution	TP	TC	CPP
1	Harvard University	446	5457	12.24
2	Shanghai Jiao Tong University	440	4056	9.22
3	University of California System	408	6018	14.75
4	Chinese Academy of Sciences	346	5826	16.84
5	National University of Singapore	289	2591	8.97
6	University of London	289	3622	12.53
7	University of Michigan System	245	2398	9.79
8	Sichuan University	338	3792	11.22
9	Pennsylvania Commonwealth System of Higher Education Pcshe	238	3222	13.54
10	University of Texas System	230	2843	12.36

The ranking is based on the number of total publications; TP = Total publications; TC = Total citing articles; CPP = Citations per publication.

## Supplementary Materials

The following are available online at https://www.mdpi.com/1996-1944/13/13/2891/s1, Table S1. Table containing all the terms visualized in the term map of topic set 1 from 1990 through 1999 with their respective occurrence frequencies, clusters and averaged citations. Table S2. Table containing all the terms visualized in the term map of topic set 1 from 2000 through 2009 with their respective occurrence frequencies, clusters and averaged citations. Table S3. Table containing all the terms visualized in the term map of topic set 1 from 2010 through 2019 with their respective occurrence frequencies, clusters and averaged citations. Table S4. Table containing all the terms visualized in the term map of topic set 2 from 1990 through 1999 with their respective occurrence frequencies, clusters and averaged citations. Table S5. Table containing all the terms visualized in the term map of topic set 2 from 2000 through 2009 with their respective occurrence frequencies, clusters and averaged citations. Table S6. Table containing all the terms visualized in the term map of topic set 2 from 2010 through 2019 with their respective occurrence frequencies, clusters and averaged citations. Table S7. Table containing all the terms visualized in the term map of topic set 3 from 1990 through 1999 with their respective occurrence frequencies, clusters and averaged citations. Table S8. Table containing all the terms visualized in the term map of topic set 3 from 2000 through 2009 with their respective occurrence frequencies, clusters and averaged citations. Table S9. Table containing all the terms visualized in the term map of topic set 3 from 2010 through 2019 with their respective occurrence frequencies, clusters and averaged citations. Figure S1. Term map for topic set 1 years 2000 through 2009. Term map showing the visualization of 287 terms. Table S2 contains all the terms visualized with their respective occurrence frequencies and averaged citations. Figure S2. Term map for years (a) 1990 through 1999 (b) 2000 through 2009 (c) 2010 through 2019. (a) Term map showing the visualization of 25 terms. Table S4 contains all the terms visualized with their respective occurrence frequencies and averaged citations. (b) Term map showing the visualization of 102 terms. Table S5 contains all the terms visualized with their respective occurrence frequencies and averaged citations. (c) Term map showing the visualization of 365 terms. Table S6 contains all the terms visualized with their respective occurrence frequencies and averaged citations. Figure S3. Term map for topic set 3 for years (a) 1990 through 1999 (b) 2000 through 2009 (a) Term map showing the visualization of 28 terms. Table S7 contains all the terms visualized with their respective occurrence frequencies and averaged citations. (b) Term map showing the visualization of 53 terms. Table S8 contains all the terms visualized with their respective occurrence frequencies and averaged citations.

## Abbreviations

aECM	Artificial Extracellular Matrix
ECM	Extra cellular matrix
TE	Tissue engineering
RM	Regenerative medicine
ts	Topic search
SCI	Spinal cord industry
MIT	Massachusetts Institute of Technology
