# Supplementary material for: A Bibliometric Review of Artificial Extracellular Matrices Based on Tissue Engineering Technology Literature: 1990 through 2019
Source: Materials (Basel). 2020 Jun 27;13(13):2891. doi: 10.3390/ma13132891 (PMC7372414; doi:10.3390/ma13132891)
Supplement: Supplementary file 1 [file materials-13-02891-s001.pdf]

materials

MDPI

Supplementary Materials

A Bibliometric Review of Artificial Extracellular Matrices Based on Tissue Engineering Technology Literature: 1990 through 2019

Pilar Simmons [1,2,3,], Taylor McElroy [1,2,3] and Antiño R. Allen [1,2,3,*]

Division of Radiation Health, University of Arkansas for Medical Sciences, Little Rock, AR 72205, USA; TMMCELROY@uams.edu

[2] Department of Pharmaceutical Sciences, University of Arkansas for Medical Sciences, Little Rock, AR 72205, USA

[3] Department of Neurobiology & Developmental Sciences, University of Arkansas for Medical Sciences, Little Rock, AR 72205, USA

* Correspondence: aallen@uams.edu Tel.: +1-501-686-7553

Received: 18 May 2020; Accepted: 24 June 2020; Published: 27 June 2020

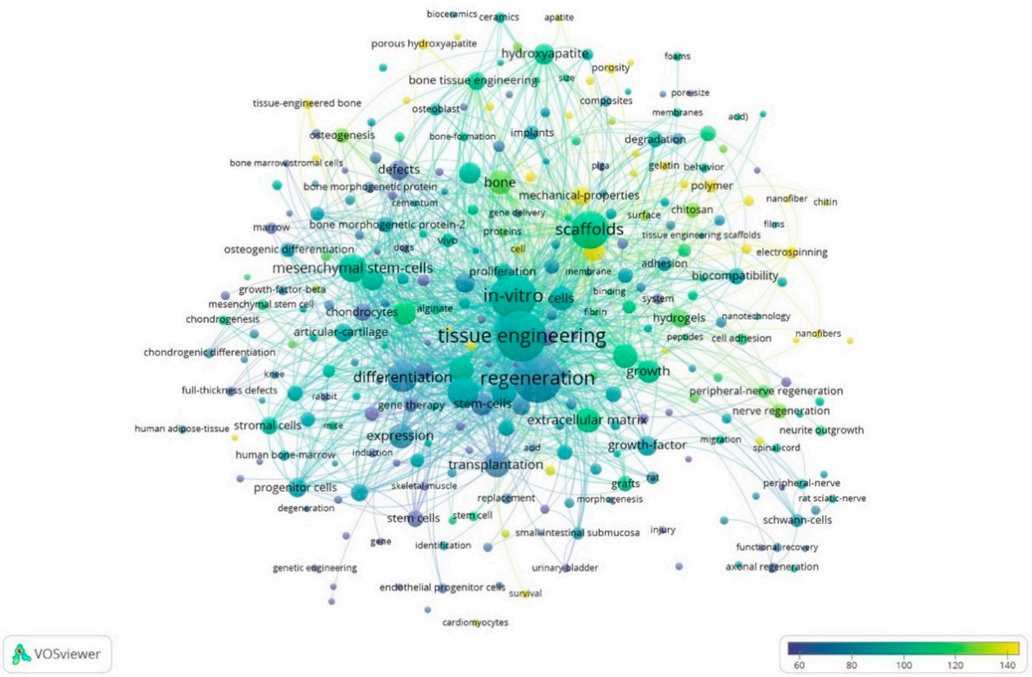

Figure S1. Term map for topic set 1 years 2000 through 2009. Term map showing the visualization of 287 terms. Table S2 contains all the terms visualized with their respective occurrence frequencies and averaged citations.

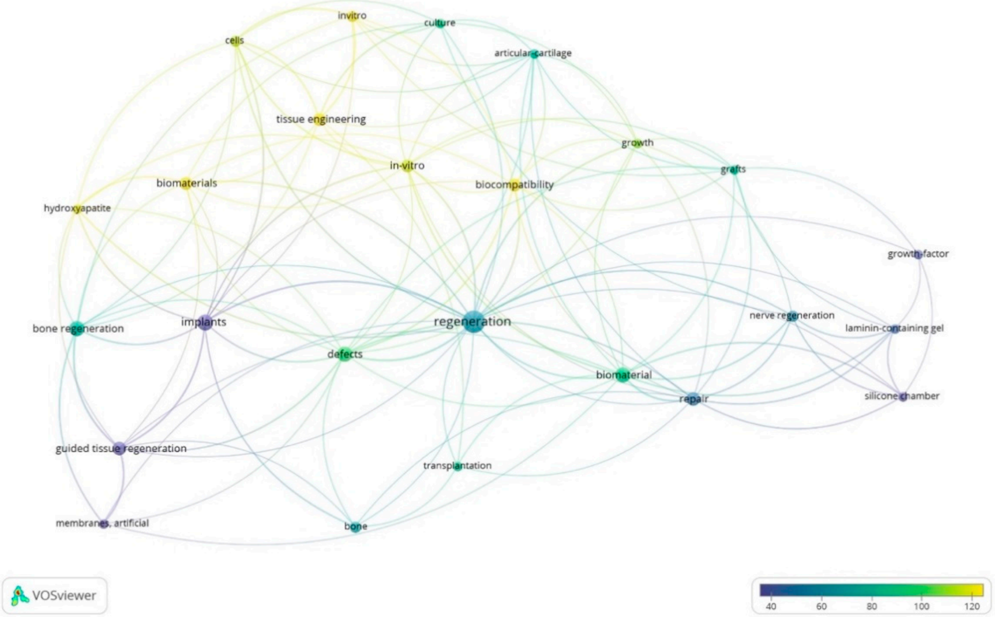
invitro
culture
cells
articular-cartilage
tissue engineering
growth
in-vitro
grafts
biomaterials
biocompatibility
hydroxyapatite
growth-factor
bone regeneration
implants
regeneration
nerve regeneration
laminin-containing gel
defects
biomaterial
repair
silicone chamber
guided tissue regeneration
transplantation
membranes, artificial
bone
VOSviewer
40
60
80
100
120

(**a**)

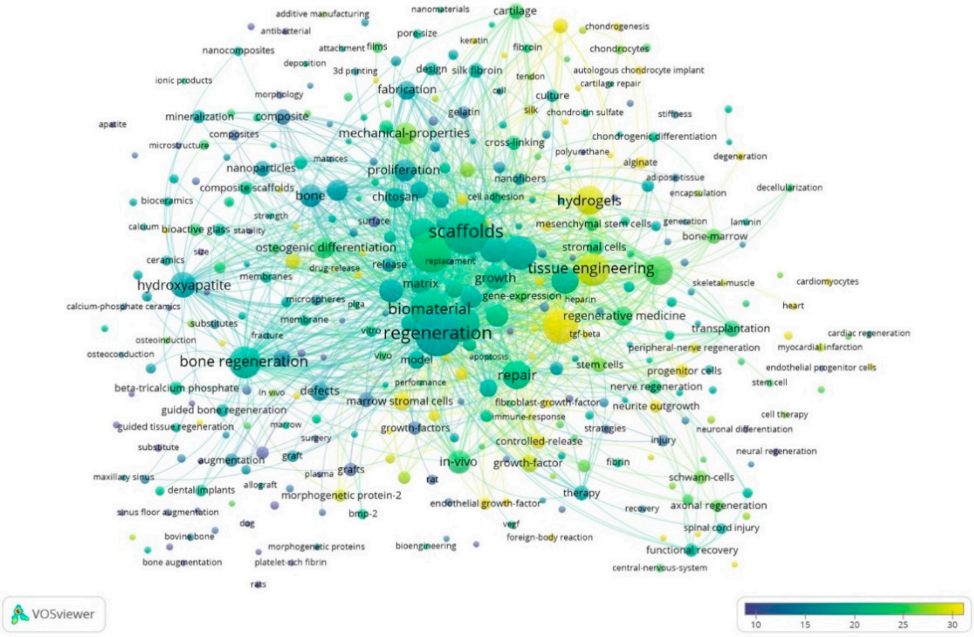
scaffolds
tissue engineering
hydrogels
biomaterial
regeneration
bone regeneration
hydroxyapatite
osteogenic differentiation
proliferation
chitosan
bone
nanoparticles
composite
mechanical-properties
fabrication
cartilage
silk fibroin
mineralization
repair
growth
matrix
regenerative medicine
transplantation
stromal cells
mesenchymal stem cells
stem cells
nerve regeneration
neurite outgrowth
marrow stromal cells
growth-factors
growth-factor
in-vivo
controlled-release
defects
axonal regeneration
schwann-cells
functional recovery
morphogenetic protein-2
dental implants
augmentation
therapy
bone-marrow
progenitor cells
VOSviewer
10
15
20
25
30

(**b**)

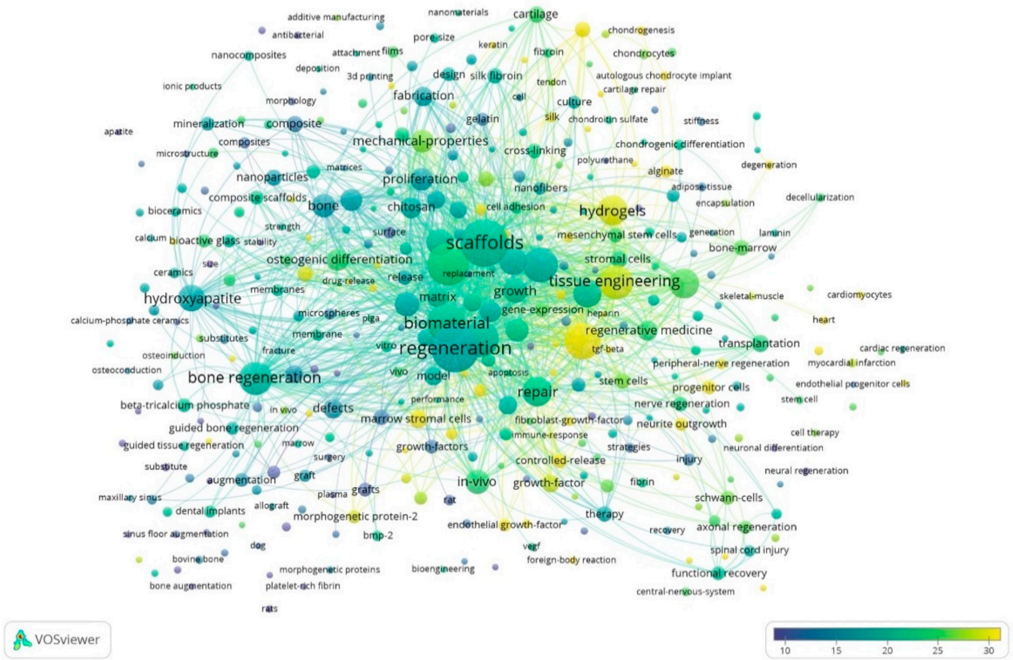

(**c**)

Figure S2. Term map for years (**a**) 1990 through 1999 (**b**) 2000 through 2009 (**c**) 2010 through 2019. (**a**) Term map showing the visualization of 25 terms. Table S4 contains all the terms visualized with their respective occurrence frequencies and averaged citations. (**b**) Term map showing the visualization of 102 terms. Table S5 contains all the terms visualized with their respective occurrence frequencies and averaged citations. (**c**) Term map showing the visualization of 365 terms. Table S6 contains all the terms visualized with their respective occurrence frequencies and averaged citations.

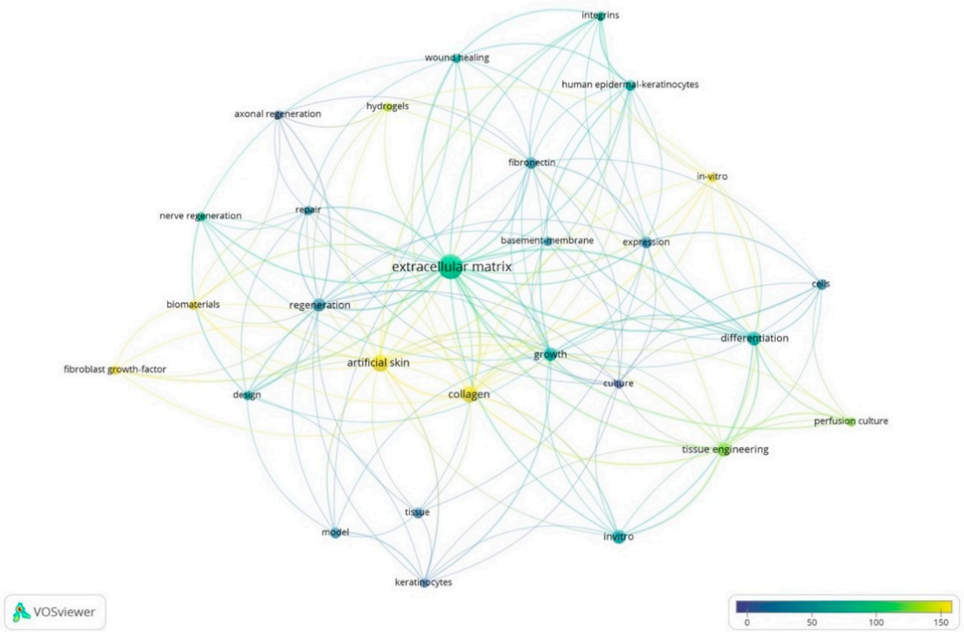

(**a**)

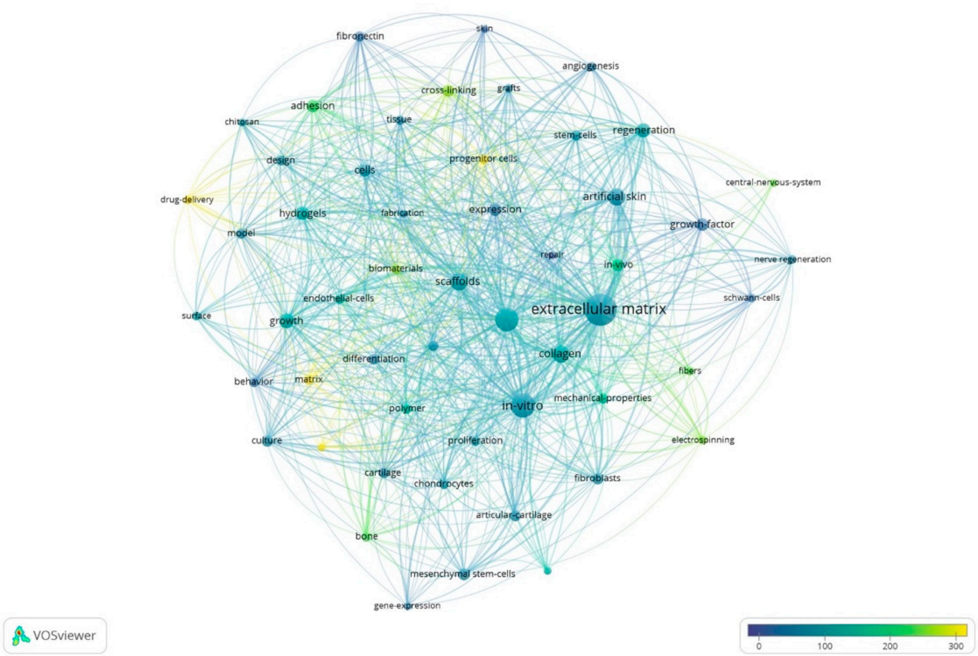

(**b**)

Figure S3. Term map for topic set 3 for years (**a**) 1990 through 1999 (**b**) 2000 through 2009 (**a**) Term map showing the visualization of 28 terms. Table S7 contains all the terms visualized with their respective occurrence frequencies and averaged citations. (**b**) Term map showing the visualization of 53 terms. Table S8 contains all the terms visualized with their respective occurrence frequencies and averaged citations.

Table S1. Table containing all the terms visualized in the term map of topic set 1 from 1990 through 1999 with their respective occurrence frequencies, clusters and averaged citations.

Label	Cluster	Occurrences	Avg. Citations
agrobacterium	2	6	41
agrobacterium-tumefaciens	2	9	32
articular-cartilage	1	7	218
beta-glucuronidase	2	6	41
biodegradable	1	5	42
biodegradable polymer scaffolds	1	7	241
biodegradable polymers	1	5	329
biomaterials	3	9	126
bone	1	8	149
bone regeneration	1	9	166
cartilage	1	10	173
cell transplantation	1	7	198
cells	1	18	96
chondrocytes	1	9	179
collagen	1	9	89
culture	1	11	135
defects	3	8	129
degradation	1	5	157
differentiation	3	9	62
dna	2	7	87
expression	2	15	52
extracellular matrix	1	16	210
gene	2	5	66
genetic engineering	2	11	40
grafts	3	6	61
growth	3	9	826
in-vitro	3	11	114
invitro	1	22	436

invivo	1	7	184
matrix	1	5	137
microprojectile bombardment	2	6	22
myoblasts	3	5	1517
nerve regeneration	1	5	109
plant-regeneration	2	9	19
plants	2	8	36
polymer	1	5	157
polymers	1	5	156
protoplasts	2	5	71
reconstruction	3	5	40
regeneration	2	41	99
repair	1	16	102
resistance	2	7	58
scaffolds	1	8	169
somatic embryogenesis	2	10	39
tissue	1	14	72
tissue culture	2	10	17
tissue engineering	1	39	169
tissue regeneration	1	5	107
transformation	2	12	47
transgenic plants	2	12	26
transplantation	1	14	572

Table S2. Table containing all the terms visualized in the term map of topic set 1 from 2000 through 2009 with their respective occurrence frequencies, clusters and averaged citations.

Table S3. Table containing all the terms visualized in the term map of topic set 1 from 2010 through 2019 with their respective occurrence frequencies, clusters and averaged citations.

Label	Cluster	Occurrences	Avg. Citations
3-dimensional scaffolds	1	51	32
3d	4	41	18
3d bioprinting	4	60	13
3d printing	1	194	19
3d scaffold	1	41	23
3d scaffolds	1	39	18
acellular dermal matrix	2	39	15
acellular matrix	2	47	18
achilles-tendon	3	43	32
acid	1	126	21
acid)	1	73	26
activation	2	177	18
acute myocardial-infarction	2	73	28
additive manufacturing	1	74	18
adhesion	1	374	21
adipogenesis	2	55	12
adipose tissue	2	47	27
adipose-derived stem cells	2	114	19
adipose-tissue	2	224	23
adsorption	1	63	15
adult	2	46	25
adult stem cells	2	46	30
alginate	4	211	29
alginate hydrogels	4	74	28
alignment	5	117	24
alkaline-phosphatase	3	38	30
allograft	3	52	14
allografts	5	43	22
amniotic membrane	2	49	15
angiogenesis	2	763	23
animal model	3	46	27
animal-models	3	71	19
annulus fibrosus	4	45	22

anterior cruciate ligament	4	123	36
antibacterial	1	68	15
antibacterial activity	1	60	26
apatite	1	55	18
apatite formation	1	35	28
apoptosis	2	92	20
architecture	1	105	23
articular cartilage	4	68	26
articular chondrocytes	4	70	28
articular-cartilage	4	561	23
articular-cartilage defects	4	41	26
articular-cartilage repair	4	48	27
attachment	1	62	19
augmentation	3	130	16
autogenous bone	3	42	18
autologous chondrocyte implantation	4	130	31
axon regeneration	5	37	15
axonal regeneration	5	117	29
bacterial cellulose	1	65	23
basement-membrane	2	42	26
behavior	1	248	26
beta-catenin	2	37	17
beta-tricalcium phosphate	3	193	31
binding	3	57	24
bioactive glass	1	250	26
bioactive glass scaffolds	1	52	32
bioactive glasses	1	47	33
bioactivity	1	146	23
bioceramics	1	111	20
biocompatibility	1	599	19
biodegradable	1	50	44
biodegradable polymers	1	75	35
biodegradation	1	79	25
bioengineering	2	99	13
biofabrication	4	68	21
bioglass	1	42	33
biologic scaffolds	2	41	22
biological-properties	1	44	41
biology	3	84	21
biomaterial	1	235	31
biomaterials	1	1457	24
biomechanics	4	54	24
biomedical applications	1	320	29
biomimetic	1	89	27
biomimetic materials	1	35	25
biomineralization	1	68	20
bioprinting	4	64	27
bioreactor	2	100	21
bioreactors	2	43	15
bladder	2	41	17
blends	1	48	16
blood	2	50	20
blood-vessels	2	82	24
bmp-2	3	175	19
bmscs	3	47	11
bombyx-mori silk	1	45	49
bone	1	1013	23
bone defect	3	60	16
bone formation	3	54	16
bone healing	3	36	21
bone marrow	2	59	21
bone marrow mesenchymal stem cells	5	66	9
bone marrow stromal cells	3	58	21
bone morphogenetic protein	3	63	33
bone morphogenetic protein-2	3	271	30

bone morphogenetic proteins	3	68	24
bone regeneration	3	1327	19
bone repair	3	88	31
bone tissue	1	49	13
bone tissue engineering	1	878	24
bone tissue regeneration	1	65	27
bone-formation	3	117	24
bone-marrow	2	616	22
bone-marrow-cells	2	52	60
brain	5	41	13
calcium	1	75	18
calcium phosphate	3	73	25
calcium phosphate cement	3	36	24
calcium-phosphate	1	202	28
calcium-phosphate cement	3	57	48
calcium-phosphate ceramics	3	55	18
calvarial defects	3	71	26
cancellous bone	3	39	30
cancer	2	83	13
capacity	2	35	15
carbon nanotubes	1	113	28
cardiac regeneration	2	53	24
cardiac tissue engineering	2	89	33
cardiomyocytes	2	111	35
cartilage	4	613	21
cartilage regeneration	4	161	20
cartilage repair	4	81	18
cartilage tissue engineering	4	182	21
cell	1	147	22
cell adhesion	1	195	33
cell culture	1	64	19
cell differentiation	2	59	23
cell encapsulation	4	43	43
cell migration	5	41	20
cell proliferation	1	60	23
cell sheet	2	72	14
cell therapy	2	153	24
cell transplantation	2	69	20
cell-culture	2	36	29
cell-proliferation	1	39	25
cells	1	881	21
cellulose	1	48	14
cement	3	39	20
central-nervous-system	5	60	25
ceramic scaffolds	1	56	50
ceramics	1	121	26
challenges	4	42	29
chemistry	1	35	18
chitin	1	57	21
chitosan	1	682	22
chondrocyte	4	93	31
chondrocytes	4	337	20
chondrogenesis	4	235	19
chondrogenic differentiation	4	359	23
chondroitin sulfate	4	104	24
clinical-applications	2	45	21
co-culture	2	65	21
coatings	1	53	21
coculture	2	72	13
collagen	1	1009	24
collagen scaffold	3	42	16
collagen scaffolds	1	77	18
colony-stimulating factor	2	36	30
combination	3	67	20
complex	2	40	31

composite	1	366	23
composite nanofibers	1	58	26
composite scaffold	1	120	18
composite scaffolds	1	485	25
composites	1	210	24
compression	4	35	15
conduits	5	101	18
constructs	4	164	18
controlled release	1	99	26
controlled-release	1	366	33
copolymer	1	35	30
copolymers	1	45	19
cornea	2	39	17
cross-linking	1	312	25
crosslinking	1	37	21
culture	2	421	21
cultures	6	57	25
cytocompatibility	1	85	15
cytokines	2	39	20
cytotoxicity	1	83	12
decellularization	2	208	21
defect	3	86	12
defects	3	481	18
degeneration	4	67	25
degradation	1	461	22
delivery	1	772	20
density	4	35	15
dental implants	3	52	17
dental pulp	2	40	20
dental pulp stem cells	2	58	18
dental-pulp	2	55	27
deposition	1	68	26
design	1	409	22
dexamethasone	1	59	21
diameter	1	46	24
differentiation	2	2457	22
directed differentiation	2	39	20
disease	2	82	16
distraction osteogenesis	3	43	25
drug	1	38	17
drug delivery	1	239	43
drug-delivery	1	589	24
drug-delivery systems	1	37	35
drug-release	1	53	22
dual delivery	3	35	32
efficacy	3	35	17
elasticity	1	42	22
elastin	1	43	24
electrical stimulation	5	46	28
electrical-stimulation	5	118	24
electrospinning	1	634	27
electrospun	1	87	28
electrospun nanofibers	5	155	27
electrospun scaffolds	1	57	24
embryonic stem-cells	2	109	51
encapsulation	4	54	24
endochondral ossification	4	61	20
endothelial cells	2	67	26
endothelial growth-factor	2	260	40
endothelial progenitor cells	2	143	27
endothelial-cells	2	309	27
engineered bone	3	38	28
engineered cartilage	4	65	21
engineering applications	1	59	25
engraftment	2	38	27

enhancement	3	45	16
epidermal-growth-factor	1	38	25
epithelial-cells	2	93	15
epsilon-caprolactone	1	48	16
exfoliated deciduous teeth	2	46	28
expansion	2	106	21
expression	2	962	21
extracellular matrix	2	1287	28
fabrication	1	853	19
factor delivery	3	50	24
factor-i	2	55	24
fate	2	49	26
fiber	1	51	29
fiber diameter	1	46	35
fibers	1	300	26
fibrin	2	141	22
fibrin glue	3	47	20
fibroblast	1	50	19
fibroblast-growth-factor	2	173	39
fibroblasts	2	293	22
fibroin	1	84	26
fibronectin	5	91	22
fibrous scaffolds	1	69	27
films	1	138	26
follow-up	4	78	20
fracture	3	37	34
full-thickness defects	4	36	30
functional recovery	5	136	19
functionalization	1	45	28
fusion	3	41	24
gel	1	51	21
gelatin	1	357	22
gellan gum	4	35	17
gels	1	50	22
gene	6	78	19
gene delivery	3	135	40
gene expression	2	47	25
gene therapy	3	90	29
gene-expression	2	455	25
gene-therapy	3	107	26
gene-transfer	2	39	25
generation	2	154	17
genipin	1	62	25
glass	1	51	32
glutaraldehyde	1	50	15
glycosaminoglycans	4	38	22
gold nanoparticles	1	45	21
graft	3	165	18
graft substitutes	3	55	25
grafts	5	257	21
graphene	1	64	25
graphene oxide	1	110	15
growth	5	890	22
growth factor	3	78	31
growth factors	3	174	30
growth-factor	5	617	26
growth-factor delivery	4	166	37
growth-factor-beta	4	74	38
growth-factor-i	2	49	20
growth-factors	3	276	22
guidance	5	63	23
guided bone regeneration	1	86	23
guided tissue regeneration	3	69	34
heart	2	116	37
heart-failure	2	47	20

heart-valves	2	36	31
heparin	1	89	22
hepatocyte growth-factor	2	40	26
hepatocytes	2	44	23
human adipose-tissue	2	118	26
human articular chondrocytes	4	87	29
human bone-marrow	2	140	36
human dental-pulp	2	42	23
human mesenchymal stem cells	3	54	25
human osteoblasts	1	50	37
hyaluronan	4	50	27
hyaluronic acid	4	117	24
hyaluronic-acid	4	255	28
hyaluronic-acid hydrogels	4	60	34
hybrid scaffolds	1	49	29
hydrogels	4	1237	25
hydroxyapatite	1	839	21
hydroxyapatite scaffolds	1	42	19
hypertrophy	4	40	20
hypoxia	2	68	23
identification	2	73	22
immobilization	1	107	18
immunomodulation	2	43	28
implant	3	51	14
implantation	4	141	22
implants	3	123	24
in vitro	6	39	17
in vivo	3	68	24
in-vitro	1	3337	26
in-vitro bioactivity	1	58	57
in-vitro characterization	1	39	27
in-vitro chondrogenesis	4	67	44
in-vitro degradation	1	101	47
in-vitro evaluation	1	80	53
in-vivo	3	881	26
in-vivo evaluation	1	54	47
induced pluripotent stem cells	2	69	19
induction	2	157	22
infiltration	1	40	25
inflammation	2	148	22
inhibition	2	54	13
injectable	4	43	28
injectable hydrogel	4	46	16
injectable hydrogels	4	51	30
injection	2	46	16
injury	5	179	18
integration	4	37	24
intervertebral disc	4	93	21
intervertebral disc degeneration	4	36	15
intraarticular injection	4	38	19
ionic products	1	48	38
keratinocytes	2	73	18
knee	4	115	22
laminin	5	65	20
left-ventricular function	2	54	30
ligament	2	59	24
linking	1	44	14
liver	2	56	15
long-term	2	36	25
low-back-pain	4	42	25
lung	2	40	24
macrophage phenotype	2	45	32
macrophages	2	69	22
management	2	38	15
marrow	3	239	19

marrow stromal cells	3	702	33
matrices	1	93	28
matrix	2	651	23
maturation	2	44	18
mechanical properties	1	160	23
mechanical stimulation	2	67	19
mechanical-properties	1	1025	27
mechanism	1	56	14
mechanisms	2	132	16
mechanobiology	2	37	14
mechanotransduction	2	64	18
medicine	2	76	23
membrane	1	120	19
membranes	1	191	20
meniscus	4	66	22
mesenchymal stem cell	2	174	24
mesenchymal stem cells	2	626	23
mesenchymal stem-cells	4	2531	28
mesenchymal stromal cells	2	207	18
metabolism	2	35	18
mice	2	99	23
microenvironment	2	49	15
microfracture	4	49	20
microparticles	1	51	18
microspheres	1	202	19
microstructure	1	65	31
migration	2	185	24
mineralization	1	300	22
model	2	525	19
models	2	40	12
modulation	2	53	19
molecular-weight	1	51	30
morphogenesis	2	71	23
morphogenetic protein-2	3	276	30
morphogenetic proteins	3	81	22
morphology	1	165	20
mouse	2	82	27
mouse model	2	51	26
mscs	2	41	15
muscle	2	116	23
muscle regeneration	2	41	33
myocardial infarction	2	84	27
myocardial regeneration	2	36	16
myocardial-infarction	2	153	33
nano-hydroxyapatite	1	95	26
nanocomposite	1	129	22
nanocomposite scaffolds	1	38	47
nanocomposites	1	186	24
nanofiber	1	97	34
nanofiber scaffolds	5	43	28
nanofibers	1	508	24
nanofibrous scaffold	1	58	20
nanofibrous scaffolds	1	207	24
nanohydroxyapatite	1	56	19
nanomaterials	1	79	18
nanoparticles	1	450	19
nanotechnology	1	87	27
nanotopography	5	49	26
necrosis-factor-alpha	2	40	23
neovascularization	2	66	24
nerve growth-factor	5	43	22
nerve regeneration	5	260	23
nerve tissue engineering	5	76	29
networks	1	66	23
neural differentiation	5	36	18

neural regeneration	5	63	10
neural stem-cells	5	83	19
neural tissue engineering	5	73	35
neurite outgrowth	5	197	38
neuronal differentiation	5	59	14
neurons	5	85	20
neurotrophic factor	5	63	23
neurotrophic factors	5	38	29
niche	2	46	25
nucleus pulposus	4	77	23
nucleus pulposus cells	4	43	28
of-the-art	1	95	44
optimization	6	35	13
organization	1	38	14
orientation	5	70	21
orthotopic transplantation	2	62	29
osseointegration	3	45	13
osteoarthritis	4	164	18
osteoblast	1	142	24
osteoblast differentiation	3	184	28
osteoblast-like cells	1	86	36
osteoblastic differentiation	3	86	22
osteoblasts	3	278	26
osteochondral defects	4	73	31
osteogenesis	3	548	20
osteogenic differentiation	3	1082	22
osteoinduction	3	56	29
osteopontin	3	41	13
osteoporosis	3	42	21
oxidative stress	2	54	16
particles	1	62	20
pathway	2	48	14
pcl	1	74	16
peg hydrogels	4	35	26
peptide	1	90	18
peptides	1	44	38
performance	1	66	22
perfusion	2	37	17
periodontal ligament	2	45	23
periodontal regeneration	2	126	25
periodontal-ligament	2	65	25
periosteum	3	53	18
peripheral nerve	5	35	17
peripheral nerve injury	5	42	19
peripheral nerve regeneration	5	68	34
peripheral-nerve	5	55	31
peripheral-nerve regeneration	5	161	46
permeability	1	35	19
phenotype	2	120	23
phosphate	1	101	18
physical-properties	1	42	28
plant-regeneration	6	45	7
plasma	1	36	12
platelet-rich plasma	3	332	28
platform	2	43	24
plga	5	125	24
pluripotent stem-cells	2	154	18
polarization	2	35	25
poly(epsilon-caprolactone)	1	105	18
poly(ethylene glycol)	4	78	27
poly(l-lactic acid)	1	50	23
poly(lactic acid)	1	35	32
poly(lactic-co-glycolic acid)	1	40	26
poly(vinyl alcohol)	1	41	36
polycaprolactone	1	272	21

polycaprolactone scaffolds	1	49	40
polymer	1	168	29
polymer nanofibers	1	46	37
polymer scaffolds	1	87	29
polymeric scaffolds	1	36	44
polymerization	1	36	24
polymers	1	200	26
polypyrrole	5	53	27
polyurethane	1	92	19
pore-size	1	212	32
porosity	1	190	22
porous hydroxyapatite	1	55	25
porous scaffold	1	63	28
porous scaffolds	1	146	32
precursor cells	2	40	28
progenitor cells	2	466	31
progenitors	2	49	24
proliferation	1	1044	20
promotes	2	53	13
protein	1	154	21
protein adsorption	1	70	32
proteins	1	111	21
pulp	2	35	18
rabbit	3	55	11
rabbit model	4	55	17
rapid prototyping	1	36	56
rat	5	161	17
rat model	2	92	16
rat sciatic-nerve	5	55	41
rats	5	75	19
recellularization	2	60	19
receptor	2	58	13
reconstruction	3	455	17
recovery	5	60	16
recruitment	2	39	16
regeneration	2	4001	20
regenerative medicine	2	721	29
release	1	344	18
repair	3	1483	21
replacement	2	130	16
resorption	3	40	13
responses	1	64	16
rgd	5	36	22
rhbmp-2	3	89	23
satellite cells	2	89	29
scaffold design	1	50	22
scaffolds	1	3710	22
schwann cell	5	39	23
schwann cells	5	74	17
schwann-cells	5	205	23
sciatic-nerve	5	56	31
self-assembly	4	53	41
self-renewal	2	80	47
shear-stress	2	48	13
sheep	3	35	12
sheets	2	45	19
signaling pathway	3	41	19
silica	1	35	14
silk	1	91	34
silk fibroin	1	304	21
silver nanoparticles	1	43	20
simulated body-fluid	1	37	27
size	1	98	19
skeletal muscle	2	55	15
skeletal-muscle	2	136	24

skin	2	189	19
skin regeneration	2	63	19
skin tissue engineering	1	59	19
small-intestinal submucosa	2	158	30
smooth-muscle	2	69	33
smooth-muscle-cells	2	150	34
sol-gel	1	46	23
somatic embryogenesis	6	39	10
spinal cord injury	5	76	20
spinal-cord	5	91	33
spinal-cord-injury	5	131	19
stability	1	41	17
stem cell	2	194	26
stem cell therapy	2	41	23
stem cells	2	593	23
stem-cell	2	40	14
stem-cell differentiation	5	41	32
stem-cells	2	1496	22
stem/progenitor cells	2	66	39
stiffness	1	125	17
stimulation	5	139	27
strategies	5	178	15
strength	1	57	29
stromal cells	2	839	24
strontium	1	51	18
subchondral bone	4	42	25
substitute	3	46	21
substitutes	3	81	21
substrate	1	35	27
surface	1	227	20
surface modification	1	268	23
surfaces	1	101	27
surgery	3	70	14
survival	2	115	21
sustained-release	1	74	33
system	5	181	17
systems	1	64	20
technology	2	35	37
teeth	2	51	21
temperature	1	57	14
tendon	2	107	21
tgf-beta	4	108	22
therapy	2	392	22
tissue	2	1522	20
tissue engineering	2	3397	22
tissue engineering applications	1	300	27
tissue engineering scaffolds	1	119	35
tissue regeneration	2	602	24
tissue scaffolds	2	36	22
tissue-engineered bone	3	152	23
tissue-engineered cartilage	4	62	17
tissue-engineered skin	2	46	25
tissue-engineering applications	4	46	32
tissue-engineering scaffolds	1	57	30
tissue-repair	2	38	21
tissues	2	65	17
titanium	1	77	18
tooth	2	45	22
tooth regeneration	2	36	21
topography	5	68	17
trachea	2	42	9
transforming growth-factor-beta-1	4	50	27
transplantation	2	823	25
tricalcium phosphate	3	123	20
umbilical-cord	2	59	27

umbilical-cord blood	2	82	24
urinary-bladder	2	38	26
vascular endothelial growth factor	3	42	20
vascular graft	1	45	23
vascular grafts	2	84	28
vascularization	2	297	20
vegf	3	148	25
viability	2	45	17
vitro	3	194	25
vivo	6	264	26
wound healing	2	225	24
wound repair	2	47	21

Table S4. Table containing all the terms visualized in the term map of topic set 2 from 1990 through 1999 with their respective occurrence frequencies, clusters and averaged citations.

Label	Cluster	Occurrences	Avg. Citations
articular-cartilage	1	3	80
biocompatibility	1	5	127
biomaterial	3	7	89
biomaterials	2	5	140
bone	4	4	68
bone regeneration	2	7	78
cells	1	4	113
culture	1	3	88
defects	2	7	95
grafts	3	3	84
growth	1	3	106
growth-factor	3	3	43
guided tissue regeneration	2	6	30
hydroxyapatite	2	3	117
implants	2	8	40
in-vitro	1	5	114
invitro	1	4	119
laminin-containing gel	3	3	48
membranes, artificial	2	3	20
nerve regeneration	3	4	59
regeneration	2	15	61
repair	3	6	52
silicone chamber	3	3	14
tissue engineering	1	5	179
transplantation	4	3	86

Table S5. Table containing all the terms visualized in the term map of topic set 2 from 2000 through 2009 with their respective occurrence frequencies, clusters and averaged citations.

Label	Cluster	Occurrences	Avg. Citations
adhesion	3	14	65.4286
alginate	5	14	96.5
angiogenesis	2	14	95.9286
articular-cartilage	5	12	249.25
attachment	2	12	61.6667
augmentation	1	10	29.3
axonal regeneration	2	14	74.7857
bio-oss	1	10	44.2
biocompatibility	3	42	66.2619
biomaterial	2	110	68.0909
biomaterial carriers	1	16	73.625
biomaterials	3	63	110.5238
bone	4	58	123.0345
bone morphogenetic protein-2	1	20	110.85
bone regeneration	1	57	56.8421
bone tissue engineering	4	12	81.0833
bone-formation	1	10	179.5

cartilage	5	18	65.7778
cell adhesion	3	15	37.4
cells	6	42	82.2857
chitin	3	10	157.3
chitosan	3	29	136.6897
chondrocytes	5	14	65.5714
collagen	3	62	70.8226
composite	3	14	61.1429
composites	1	16	49.875
controlled release	1	12	149.6667
controlled-release	1	15	55.8667
culture	4	16	55.125
cytotoxicity	3	10	54
defects	1	37	51.2703
degradation	4	21	50.4762
delivery	3	25	53.6
dental implants	1	15	75.8667
differentiation	4	52	62.4038
dogs	1	17	58.4118
drug-delivery	2	14	65.5714
expression	2	26	60.0385
extracellular matrix	2	42	71.3571
fabrication	3	12	155.0833
fibers	3	10	116.6
fibroblast-growth-factor	1	13	94.6154
fibroblasts	2	15	94
fibronectin	2	14	71.8571
functional recovery	2	11	56.6364
gene-expression	4	11	140.3636
graft	6	12	53.8333
grafts	2	26	49.8462
growth	3	37	73.7568
growth factors	1	12	97.5833
growth-factor	2	28	64.5357
guided tissue regeneration	1	20	35.9
human osteogenic protein-1	1	13	62.9231
hydrogels	2	28	60.6071
hydroxyapatite	1	56	61.5357
implants	1	24	62.2083
in-vitro	4	109	97.6422
in-vivo	2	48	96.7292
invitro	6	11	53
marrow stromal cells	4	28	129.9286
matrix	5	42	78.2619
mechanical properties	3	10	44.3
mechanical-properties	3	18	109.2222
membrane	1	11	46.4545
membranes	1	16	115.875
mesenchymal stem-cells	4	33	230.3939
model	1	18	64.2222
morphogenetic protein-2	1	18	69.6667
nerve regeneration	2	17	100.3529
osteoblast	4	10	90.8
osteoblasts	4	17	51.4706
osteogenesis	4	17	275.4118
periodontal regeneration	1	12	55.6667
peripheral-nerve regeneration	2	11	173.0909
pharmacokinetics	1	12	82.25
platelet-rich plasma	1	10	68.1
polymer	2	14	63.2143
porosity	4	13	314.7692
proliferation	6	23	77.3913
rat	2	12	42.4167
reconstruction	1	24	49.1667
regeneration	1	155	67.7419

release	3	14	72.4286
repair	2	64	95.7812
rhbmp-2	1	17	38.4118
scaffolds	3	96	117.0833
schwann-cells	2	12	121.9167
small-intestinal submucosa	6	13	38.2308
spinal-cord	2	10	134.7
stem-cells	2	23	80.5652
substitutes	4	10	40.3
surface	2	14	74.0714
surfaces	3	10	37.7
surgery	1	14	42.7143
system	3	10	41.3
therapy	1	14	62.8571
tissue	1	35	86.6857
tissue engineering	2	90	160.1222
tissue regeneration	1	22	67.8636
transplantation	5	23	74.6957
vivo	4	11	99.8182
wound healing	1	18	88.7222

Table S6. Table containing all the terms visualized in the term map of topic set 2 from 2010 through 2019 with their respective occurrence frequencies, clusters and averaged citations.

Label	Cluster	Occurrences	Avg. Citations
3d printing	1	28	15
acid	1	44	18
activation	3	69	16
acute myocardial-infarction	3	23	25
additive manufacturing	1	15	26
adhesion	1	108	18
adipose-derived stem cells	2	16	13
adipose-tissue	5	32	14
adsorption	1	33	14
adult-rat	4	16	42
alginate	5	58	31
alginate hydrogels	5	16	21
alignment	4	20	14
allograft	2	16	17
angiogenesis	3	125	19
animal-models	2	21	18
anterior cruciate ligament	5	16	34
antibacterial	1	22	11
apatite	1	15	11
apoptosis	3	19	22
architecture	1	20	25
articular-cartilage	5	89	30
attachment	1	17	19
augmentation	2	72	15
autogenous bone	2	38	14
autologous chondrocyte implantation	5	31	48
axonal regeneration	4	62	25
bacterial cellulose	1	29	23
barrier membranes	2	15	9
behavior	1	75	21
beta-tricalcium phosphate	2	69	21
binding	3	21	25
bioactive glass	1	63	24
bioactivity	1	42	22
bioceramics	1	39	20
biocompatibility	1	172	17
biodegradation	1	26	19
bioengineering	4	17	21
biomaterial	1	523	21

biomaterial scaffolds	1	46	18
biomaterials	3	482	20
biomedical applications	1	83	23
biomimetic	1	17	20
biomineralization	1	18	13
bioreactor	5	17	21
biphasic calcium-phosphate	2	20	25
bmp-2	2	40	21
bone	1	210	16
bone augmentation	2	16	3
bone graft	2	29	14
bone morphogenetic protein-2	2	38	29
bone morphogenetic proteins	2	16	20
bone regeneration	2	404	20
bone repair	2	35	16
bone substitute	2	20	7
bone substitutes	2	35	21
bone tissue	1	15	14
bone tissue engineering	1	86	29
bone-formation	1	34	18
bone-marrow	5	88	25
bovine bone	2	25	15
brain	4	15	14
calcium	1	23	18
calcium phosphate	2	34	18
calcium-phosphate	1	56	24
calcium-phosphate cement	1	15	31
calcium-phosphate ceramics	2	19	13
carbon nanotubes	1	21	28
cardiac regeneration	3	17	19
cardiomyocytes	3	22	32
cartilage	5	95	24
cartilage regeneration	5	30	24
cartilage repair	5	18	21
cell	6	27	17
cell adhesion	1	55	22
cell therapy	3	21	26
cell-proliferation	1	17	14
cells	1	219	17
central-nervous-system	4	29	24
ceramics	1	44	19
chemistry	1	17	22
chitin	3	22	22
chitosan	1	162	18
chondrocytes	5	34	26
chondrogenesis	5	31	30
chondrogenic differentiation	5	45	21
chondroitin sulfate	5	26	21
coatings	1	18	18
collagen	5	263	21
combination	2	33	10
composite	1	97	13
composite multilayered biomaterial	5	16	24
composite scaffold	1	24	15
composite scaffolds	1	74	22
composites	1	50	12
conduits	4	26	14
controlled release	3	30	19
controlled-release	3	83	45
cross-linking	1	82	22
culture	6	62	18
cytocompatibility	1	26	24
cytokines	3	15	16
cytotoxicity	1	26	14
decellularization	6	32	23

defects	2	136	15
degeneration	6	30	32
degradation	1	118	18
delivery	3	188	21
dental implant	2	15	25
dental implants	2	57	21
deposition	1	16	20
derivatives	3	15	32
design	1	70	19
differentiation	3	444	19
dissolution	1	19	11
dog	2	16	12
drug delivery	1	65	66
drug-delivery	1	120	21
drug-release	1	15	29
electrical-stimulation	4	20	24
electrospinning	1	91	26
electrospun nanofibers	4	26	23
embryonic stem-cells	4	19	33
encapsulation	3	19	27
endothelial growth-factor	3	54	65
endothelial progenitor cells	3	18	12
endothelial-cells	3	48	20
enhancement	1	18	13
expansion	3	17	14
expression	3	166	18
extracellular matrix	3	325	25
extraction	2	16	11
fabrication	1	139	17
fibers	1	56	15
fibrin	3	35	21
fibroblast-growth-factor	3	37	32
fibroblasts	3	40	18
fibroin	5	34	23
fibronectin	3	23	17
fibrosis	3	16	18
films	1	47	24
follow-up	5	21	32
foreign-body reaction	3	23	30
fracture	1	15	14
functional recovery	4	70	21
gelatin	5	66	13
gene delivery	4	29	36
gene expression	2	18	9
gene-expression	3	78	25
generation	3	15	14
graft	2	57	16
graft substitutes	2	16	9
grafts	2	70	11
graphene oxide	1	20	18
growth	4	198	21
growth factors	2	46	34
growth-factor	4	119	28
growth-factor delivery	5	35	32
growth-factor-beta	3	18	55
growth-factors	2	69	12
guidance	4	15	19
guided bone regeneration	2	67	19
guided tissue regeneration	2	36	20
heart	3	27	38
heart-failure	3	18	25
heparin	3	17	31
histology	2	17	11
human bone-marrow	5	18	43
human hair	1	15	32

hyaluronic acid	5	38	22
hyaluronic-acid	5	64	18
hydrogels	3	327	29
hydroxyapatite	2	263	17
iliac crest	2	17	33
immobilization	3	20	18
immune-response	3	17	29
immunomodulation	3	27	18
implant	2	26	8
implant placement	2	19	8
implantation	2	35	18
implants	2	62	11
in vivo	2	24	27
in-vitro	1	652	23
in-vitro bioactivity	1	15	38
in-vitro degradation	1	18	44
in-vivo	2	211	24
in-vivo evaluation	4	17	48
induction	3	20	13
inflammation	3	65	26
inflammatory response	3	16	27
injury	4	53	13
intervertebral disc	6	24	21
ionic products	1	18	23
keratin	1	23	43
knee	5	28	27
laminin	6	20	19
left-ventricular function	3	15	33
local-delivery	4	16	10
macrophage	3	24	25
macrophage phenotype	3	22	24
macrophage polarization	3	21	12
macrophages	3	38	15
magnesium	1	26	12
marrow	2	26	21
marrow stromal cells	4	117	30
matrices	1	16	13
matrix	2	157	20
maxillary sinus	2	18	16
mechanical properties	1	23	26
mechanical-properties	1	196	26
mechanisms	3	32	14
membrane	2	46	21
membranes	1	58	20
mesenchymal stem cell	5	26	19
mesenchymal stem cells	5	82	24
mesenchymal stem-cells	5	485	34
mesenchymal stromal cells	3	27	14
microspheres	1	48	14
microstructure	1	20	12
migration	3	40	20
mineral trioxide aggregate	2	21	12
mineralization	1	68	18
model	2	131	19
modulation	3	19	11
morphogenetic protein-2	2	66	29
morphogenetic proteins	2	23	16
morphology	1	28	12
muscle	3	27	19
myocardial infarction	3	32	28
myocardial-infarction	3	30	48
nano-hydroxyapatite	1	21	16
nanocomposite	1	29	22
nanocomposites	1	38	19
nanofibers	1	65	17

nanofibrous scaffolds	1	31	15
nanomaterials	1	15	22
nanoparticles	1	94	16
nanotechnology	4	17	21
nerve regeneration	4	81	23
neural regeneration	4	21	7
neural stem-cells	4	44	20
neurite outgrowth	4	60	30
neuronal differentiation	4	17	10
neurons	4	29	18
neurotrophic factor	4	24	27
neurotrophic factors	4	15	31
nucleus pulposus	6	17	19
of-the-art	1	24	23
orientation	1	15	18
osseointegration	2	38	13
osteoarthritis	5	23	13
osteoblast	1	48	24
osteoblast differentiation	2	36	22
osteoblast-like cells	2	16	33
osteoblasts	1	61	20
osteoconduction	2	15	15
osteogenesis	2	117	15
osteogenic differentiation	1	175	24
osteoinduction	2	19	26
osteoporosis	2	28	26
particles	2	27	19
peptide	1	18	19
peptides	3	16	12
performance	2	15	19
periodontal regeneration	2	22	31
peripheral nerve injury	4	22	26
peripheral nerve regeneration	4	18	25
peripheral-nerve regeneration	4	41	71
phenotype	3	26	14
phosphate	2	43	16
plasma	2	15	11
platelet-rich fibrin	2	18	9
platelet-rich plasma	2	65	27
plga	1	20	22
pluripotent stem-cells	3	19	16
polarization	3	25	10
polycaprolactone	1	40	16
polymer	1	37	38
polymer scaffolds	4	17	24
polymers	1	47	21
polyurethane	5	17	11
pore-size	5	34	20
porosity	1	42	24
porous hydroxyapatite	2	19	21
porous scaffolds	1	17	31
prf	2	16	8
progenitor cells	3	69	31
proliferation	1	187	17
protein	1	51	30
protein adsorption	1	27	30
proteins	1	36	11
rabbit	2	15	7
rat	4	42	12
rat model	4	21	20
rat sciatic-nerve	4	16	35
rats	2	19	5
reconstruction	2	86	13
recovery	4	25	15
regeneration	2	840	18

regenerative medicine	3	152	24
release	1	79	17
repair	4	328	22
replacement	2	19	12
resorption	2	20	15
responses	5	23	19
rhbmp-2	2	24	13
ridge preservation	2	18	28
satellite cells	3	15	26
scaffolds	1	839	20
schwann cells	4	18	29
schwann-cells	4	69	26
self-assembly	1	18	22
silk	5	40	35
silk fibroin	1	87	19
silver nanoparticles	1	17	14
sinus augmentation	2	22	12
sinus floor augmentation	2	19	12
size	1	21	8
skeletal-muscle	3	23	28
skin	1	44	16
small-intestinal submucosa	3	37	22
smooth-muscle-cells	3	21	23
spinal cord injury	4	52	18
spinal-cord	4	24	27
spinal-cord-injury	4	47	24
stability	1	17	10
stem cell	4	24	24
stem cells	3	91	26
stem-cells	3	291	20
stiffness	3	25	14
stimulation	1	22	11
strategies	4	36	12
strength	1	19	19
stromal cells	5	106	23
strontium	1	25	18
substitute	2	24	8
substitutes	2	36	15
surface	1	59	11
surface modification	1	53	20
surfaces	1	24	41
surgery	2	23	12
survival	2	26	13
sustained-release	3	20	24
system	4	39	13
systems	1	15	21
tendon	5	22	27
tgf-beta	3	24	30
therapy	2	87	17
tissue	5	285	19
tissue engineering	5	424	28
tissue engineering applications	1	39	40
tissue regeneration	3	121	19
titanium	1	45	18
tooth extraction	2	21	21
topography	1	23	6
transplantation	4	109	23
tricalcium phosphate	2	45	16
vascular grafts	3	16	18
vascularization	3	63	22
vegf	3	24	22
vitro	4	51	21
vivo	3	45	24
wound healing	3	71	21
xenograft	2	17	4

zinc	1	16	10

Table S7. Table containing all the terms visualized in the term map of topic set 3 from 1990 through 1999 with their respective occurrence frequencies, clusters and averaged citations.

Label	Cluster	Occurrences	Avg. Citations
artificial skin	2	10	153
axonal regeneration	1	3	17
basement-membrane	1	3	39
biomaterials	1	3	271
cells	3	4	34
collagen	4	9	148
culture	1	3	14
design	2	3	53
differentiation	4	7	58
expression	3	5	35
extracellular matrix	1	21	90
fibroblast growth-factor	1	3	285
fibronectin	3	5	35
growth	2	6	60
human epidermal-keratinocytes	3	4	59
hydrogels	1	3	136
in-vitro	4	3	213
integrins	3	3	72
invitro	4	7	53
keratinocytes	2	3	20
model	2	4	36
nerve regeneration	2	3	77
perfusion culture	4	3	124
regeneration	1	6	33
repair	1	3	38
tissue	2	4	25
tissue engineering	4	7	123

Table S8. Table containing all the terms visualized in the term map of topic set 3 from 2000 through 2009 with their respective occurrence frequencies, clusters and averaged citations.

Label	Cluster	Occurrences	Avg. Citations
adhesion	1	20	212
angiogenesis	3	12	60
articular-cartilage	2	15	72
artificial skin	4	36	73
behavior	1	11	46
biomaterials	2	11	256
bone	2	12	222
cartilage	2	13	65
cells	1	20	80
central-nervous-system	3	8	234
chitosan	1	8	87
chondrocytes	2	11	87
collagen	4	37	109
cross-linking	4	18	251
culture	1	15	77
design	1	13	79
differentiation	2	14	60
drug-delivery	1	9	397
electrospinning	4	9	243
embryonic stem-cells	3	8	452
endothelial-cells	3	12	131
expression	1	19	53
extracellular matrix	3	125	77
fabrication	2	8	69
fibers	4	10	228
fibroblasts	4	16	82

fibronectin	1	14	42
gene-expression	2	8	55
grafts	5	9	65
growth	2	27	118
growth-factor	5	20	42
hydrogels	1	23	113
in-vitro	3	72	77
in-vivo	3	18	178
matrix	1	11	355
mechanical-properties	4	14	162
mesenchymal stem-cells	2	17	72
model	1	13	70
nerve regeneration	5	9	78
polymer	1	11	142
progenitor cells	3	12	366
proliferation	2	11	87
regeneration	4	26	103
repair	5	8	30
scaffolds	2	38	90
schwann-cells	5	9	39
skin	4	8	39
smooth-muscle-cells	3	8	134
stem-cells	3	14	81
surface	1	9	97
tissue	5	12	70
tissue engineering	4	70	99
transplantation	3	13	72

Table S9. Table containing all the terms visualized in the term map of topic set 3 from 2010 through 2019 with their respective occurrence frequencies, clusters and averaged citations.

Label	Cluster	Occurrences	Avg. Citations
adhesion	1	49	22
alginate hydrogels	2	14	49
angiogenesis	1	25	17
articular-cartilage	3	19	14
artificial skin	3	30	67
biocompatibility	2	22	48
biomaterial	5	16	12
biomaterials	1	88	26
biomedical applications	2	21	40
bone	4	30	29
bone regeneration	4	13	18
bone-marrow	4	23	27
cartilage	3	24	15
cell adhesion	1	28	34
cells	3	37	13
chitosan	2	18	30
chondroitin sulfate	4	20	26
collagen	3	78	31
constructs	5	14	34
cross-linking	2	21	64
culture	1	41	18
decellularization	3	27	14
delivery	2	22	24
design	2	24	17
differentiation	1	115	22
drug-delivery	2	21	53
electrospinning	2	26	34
embryonic stem-cells	5	15	30
endothelial-cells	1	30	35
expression	1	45	14
extracellular matrix	3	307	24
fabrication	2	31	22

fibroblast-growth-factor	2	16	76
fibroblasts	3	23	15
fibronectin	1	20	27
gelatin	2	22	36
gene-expression	4	25	14
glycosaminoglycans	4	15	19
growth	1	54	21
growth-factor	3	25	13
growth-factors	2	19	19
hyaluronic-acid	2	22	32
hydrogels	1	84	35
hydroxyapatite	4	15	35
immobilization	1	15	27
in-vitro	4	186	26
in-vivo	5	32	34
laminin	1	13	8
marrow stromal cells	4	16	39
matrix	3	28	28
mechanical-properties	2	59	54
mesenchymal stem cells	4	19	14
mesenchymal stem-cells	4	116	33
migration	1	21	20
model	3	32	12
nanofibers	1	17	15
nanofibrous scaffolds	2	17	29
neurite outgrowth	5	23	36
osteogenic differentiation	4	42	25
peptide	1	19	29
progenitor cells	5	27	23
proliferation	1	49	19
reconstruction	3	17	11
regeneration	3	80	21
regenerative medicine	2	41	58
release	2	14	17
repair	3	40	17
scaffolds	3	159	21
self-renewal	1	21	34
stem cells	5	24	35
stem-cells	1	80	24
stiffness	1	14	32
stromal cells	4	25	12
surface modification	2	18	28
surfaces	1	17	16
tissue	3	87	22
tissue engineering	2	150	34
transplantation	3	34	22
wound healing	3	18	24

© 2020 by the authors. Licensee MDPI, Basel, Switzerland. This article is an open access article distributed under the terms and conditions of the Creative Commons Attribution (CC BY) license (http://creativecommons.org/licenses/by/4.0/).
